# Association between serum inflammatory parameters and the disease severity in COVID‐19 patients

**DOI:** 10.1002/jcla.24162

**Published:** 2021-12-07

**Authors:** Rajab Mardani, Mehrnoush namavar, Elham ghorbi, Zabihollah Shoja, Fatemeh Zali, Hooman Kaghazian, Mohammad Reza Aghasadeghi, Seyed Amir Sadeghi, Shahram Sabeti, Ilad Alavi Darazam, Nayebali Ahmadi, Seyed Dawood Mousavi‐Nasab

**Affiliations:** ^1^ Department of Clinical Biochemistry Hamadan University of Medical Science Hamadan Iran; ^2^ Viral Vaccine Research Center Pasteur Institute of Iran Tehran; ^3^ Virology Department Pasteur Institute of Iran Tehran Iran; ^4^ Department of Clinical Biochemistry Faculty of Medicine Tehran University of Medical Science Tehran Iran; ^5^ Department of Research and Development, Production and Research Complex Pasteur Institut of Iran Tehran Iran; ^6^ Department of Hepatitis and AIDS Pasteur Institute of Iran Tehran Iran; ^7^ Pathology Ward Loghman Hakim Hospital Shahid Beheshti University of Medical Sciences Tehran Iran; ^8^ Infectious Diseases, and Tropical Research Center Loghman Hakim Hospital Shahid Beheshti University of Medical Sciences Tehran Iran; ^9^ Department of Medical Lab Technology Proteomics Research Center Faculty of Paramedical Sciences Shahid Beheshti University of Medical Sciences Tehran Iran

**Keywords:** COVID‐19, CRP, cytokines, IL‐6, SARS‐CoV‐2, TNF‐α

## Abstract

**Objective:**

Most patients infected with the novel coronavirus (SARS‐CoV‐2), as the causative agent of COVID‐19 disease, show mild symptoms, but some of them develop severe illness. The purpose of this study was to analyze the blood markers of COVID‐19 patients and to investigate the correlation between serum inflammatory cytokines and the disease severity.

**Methods:**

In this prospective cross‐sectional study, 50 patients with COVID‐19 and 20 patients without COVID‐19 were enrolled. According to ICU admission criteria, patients were divided into two groups of non‐severe and severe. Differences in the serum levels of C‐reactive protein (CRP), IL‐6, and TNF‐α, as well as erythrocyte sedimentation rate (ESR), lymphocytes (LYM) count, and neutrophils (NEU) count between the two groups were determined and analyzed.

**Results:**

Out of the 50 patients with COVID‐19, 14 were diagnosed as severe cases. There was no significant difference between the two groups of COVID‐19 patients in terms of gender and age. Blood tests of COVID‐19 patients showed a significant decrease and increase in NEU and LYM counts, respectively. There were significant differences in the serum levels of IL‐6, TNF‐α, and CRP between the severe and non‐severe groups, which were higher in the severe group.

Also, there was a significant correlation between the disease severity and CRP with ESR (*r* = 0.79), CRP with IL‐6 (*r* = 0.74), LYM with NEU (*r* = −0.97), and ESR with TNF‐α (*r* = 0.7).

**Conclusion:**

The findings of this study, as the first study in Iran, suggest that the levels of IL‐6, TNF‐α, ESR, and CRP could be used to predict the severity of COVID‐19 disease.

## Graphical abstract

Serum inflammatory parameters were analyzed in patients with severe and non‐severe COVID‐19. The serum levels of LYM, CRP, IL‐6, TNF‐α and ESR in severe patients were significantly higher than in non‐severe patients. Measurement of inflammatory markers might help clinicians monitor and evaluate the severity and prognosis of COVID‐19.

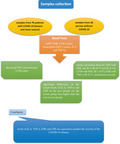

## 1 INTRODUCTION

The novel coronavirus (SARS‐CoV‐2), as the causative agent of COVID‐19 disease (coronavirus disease 2019), was first isolated on January 7, 2020, by the Chinese authorities.^1^ The disease rapidly spread throughout the world and was declared as a public health emergency of international concern by the World Health Organization (WHO) on January 30, 2020.^2^ Generally, patients with critical and severe COVID‐19 are treated in the intensive care unit (ICU), while patients with non‐severe disease are hospitalized in a usual isolation room.^1^

Studies have shown that clinical hematology parameters, such as complete blood count (CBC), play an important role in the early diagnosis of acute pulmonary diseases in patients in the triage stage during outbreaks.^2^ In addition, C‐reactive protein (CRP) level could be used in the early pneumonia diagnosis^3^ because it increases in 75%–93% of COVID‐19 cases and therefore could be used along with other biomarkers such as lymphocyte count to assess lung lesions and the severity of COVID‐19 disease.^4^

Another parameter is ESR which is widely used as an inexpensive and available test in routine laboratory patient workup, regardless of clinical questions.^5^ Several cytokine species are widely used as imaging biomarkers to describe the immune system function, predict diseases, and monitor their evolution and progression.^6^

Different types of pro‐inflammatory cytokines (IL‐1, IL‐2, IL‐6, IL‐8, and TNF‐α), produced primarily by activated macrophages, are involved in upregulating inflammatory immune responses.^7^ Like in other viral infections, NF‐μB activation via the MyD88 pathway plays a major role in the progression of COVID‐19 infection through stimulation of several pro‐inflammatory cytokines, including interleukin‐6 (IL‐6) and tumor necrosis factor alpha (TNFα).^8^ TNF‐α is a pleiotropic cytokine which is produced by activated macrophages and monocytes and regulates a variety of physiological and pathological processes.^9^ The expression of IL‐6 and TNF‐α increases and decreases during the process of illness and recovery.^10^

In COVID‐19 patients with severe symptoms and poor prognosis, IL‐6, and TNF‐α rapidly increase, while in patients with milder symptoms, these cytokines are reduced to lower levels.^11^ Moreover, some reports describing the immunological profile of COVID‐19 patients have suggested that hyper‐activation of the humoral immune system, including the secretion of interleukin (IL)‐6, is a critical mediator for respiratory failure and multi‐organ dysfunction.^12^

During the COVID‐19 pandemic, different countries have investigated and applied various approaches to the diagnosis and prognosis of COVID‐19 patients. According to previous studies on viral pneumonia as well as current clinical experience with severe COVID‐19, the storm of inflammatory factors may be the main reason for rapid disease progression and poor treatment response.^13^, ^14^ Therefore, in this study, serum inflammatory parameters were analyzed in patients with severe and non‐severe COVID‐19 to explore potential markers that allow to precisely monitor the disease progression.

## 2 STUDY DESIGN AND SETTING

### 2.1 Participants

COVID‐19 disease was diagnosed in patients based on the WHO interim guideline as described previously. In Loghman hospital in Tehran (Iran), a total of 50 patients who were diagnosed with COVID‐19 disease from March 26 to April 22/ 2020 were enrolled in this study. In addition, 20 patients without COVID‐19 disease were also enrolled as control. COVID‐19 patients were divided into two groups based on their disease severity, including the severe and non‐severe groups. The study protocol was approved by the Ethics Committee of Shahid Beheshti University of Medical Sciences.

### 2.2 Data gathering

Patients’ demographic data were gathered, including age, gender, and clinical data. Blood samples were taken from each participant, and the following blood tests were conducted on the samples, including lymphocyte count (LYM), neutrophil count (NEU), and erythrocyte sedimentation rate (ESR). In addition, C‐reactive protein (CRP) was measured using CRP immunoturbidimetric PARS Azmon kit and a HITACH 7600–020 automatic biochemical analyzer.

### 2.3 Determination of serum TNF and IL‐6 level

Anti‐TNF‐α and IL‐6 drug are option of possible therapeutic strategy for COVID‐19 patients. None of the patients used these drugs. Serum TNF‐α and IL‐6 levels in blood samples were calculated by ELISA kits (eBioscience) according to the manufacturer's instructions. The limit of detection of TNF‐α and IL‐6 was 5 and 4 pg/ml.

### 2.4 Statistical analysis

The statistical analysis was carried out using Graphpad software Version 8. Using chi‐square test, categorical variables were analyzed. Data with a normal distribution were summarized as mean ± SD and analyzed by ANOVA test. Using Spearman's rank correlation coefficient, the relationship between variables was calculated. A *p* value of <0.05 was considered as statistically significant.

## 3 RESULTS

### 3.1 Characteristics of the admitted cases

A total of 50 COVID‐19 positive (case group) and 20 COVID‐19 negative cases (control group) with a mean age of 42.7 ± 12.4 years (range: 19–78) were included in this study. The highest proportion of patients belonged to the age group of 30–49 years (40.2%). Also, 40 (57.1%) cases were male, and the rest were female. COVID‐19 positive cases included 14 (28%) patients with severe disease, admitted in ICU, and 36 (73%) patients with non‐severe disease, admitted in the emergency department.

### 3.2 Laboratory parameters

Table 1 compares patients’ laboratory parameters between negative and positive COVID‐19 cases. The serum levels of IL‐6, TNF‐α, and CRP in positive patients were significantly higher than in the negative cases.

**TABLE 1:** Gender and serum inflammatory parameters in patients with and without COVID‐19 disease

Characteristic	Control^*^, *N* = 20^1^	Case, *N* = 50^¶^	*p*‐Value^*^
Severity			0.8
Emergency	16 (80%)	36 (73%)	
ICU	4 (20%)	14 (28%)	
TNF	23 (20, 27)	66 (45, 82)	<0.001
ESR	8 (7, 10)	36 (20, 46)	<0.001
IL−6	12 (10, 13)	20 (15, 27)	<0.001
CRP	4.25 (4.47, 5.30)	8.70 (6.75, 9.70)	<0.001
NEU	55 (49, 57)	60 (57, 66)	<0.001
LYN	44 (41, 51)	37 (31, 41)	<0.001
Sex			>0.9
Female	9 (45%)	21 (42%)	
Male	11 (55%)	29 (58%)	
Age	46 (40, 51)	40 (31, 52)	0.3

Table 2 compares patients’ laboratory parameters between non‐severe and severe COVID‐19 patients. There was no significant difference in terms of gender and age between the two groups. Laboratory studies showed that 4 (28.6%) severe and 4 (5%) non‐severe cases had neutropenia, and 9 (64.3%) severe and 24 (29%) non‐severe cases had lymphopenia. There was no significant difference in the mean number of WBC and LYM between severe and non‐severe COVID‐19 patients (Table 2). In 11 (78.6%) severe and 16 (19%) non‐severe cases, the erythrocyte sedimentation rate (ESR) was high, whereas the level of ESR was significantly higher in severe patients than in non‐severe patients.

**TABLE 2:** Gender and serum inflammatory parameters in patients with severe and non‐severe COVID‐19

Characteristic	Positive Covid−19 (Case)		Control *N* = 20^¶^	*p*‐value^*^
	Emergency, *N* = 36^¶^	ICU, *N* = 14^¶^		
TNF	44 (25, 76)	54 (28, 64)	23 (20, 27)	<0.001
ESR	20 (9, 42)	31 (14, 40)	8 (7, 10)	<0.001
IL−6	15 (11, 24)	18 (13, 26)	12 (10, 13)	0.002
CRP	6.80 (5.05, 8.90)	8.90 (6.70, 9.70)	4.25 (4.47, 5.30)	0.12
NEU	60 (52, 64)	59 (57, 61)	55 (49, 57)	0.9
LYN	39 (32, 44)	40 (35, 41)	44 (41, 51)	0.7
Sex				0.4
Female	16 (44%)	6 (52.9%)	9 (45%)	
Male	20 (56%)	8 (57.1%)	11 (55%)	
Age	44 (36, 53)	38 (29, 47)	46 (40, 51)	0.2

^¶^Statistics presented: *n* (%); Median (IQR).

^*^Statistical tests performed: Fisher's exact test; ANOVA test; chi‐square test of independence.

^a^Data were expressed as ± SD for quantitative measures and also both number and percentage.

^b^Comparison using chi‐square test. Intensive Care Unit(ICU), neutrophils (NEU), lymphocyte (LYM), C‐reactive protein (CRP), ESR (Erythrocyte sedimentation rate), and TNF‐α (tumor necrosis factor alpha).

In addition, IL‐6 levels were also found to be lower than 7.4 pg/ml in non‐severe patients and higher than 8.2 pg/ml in severe patients. The serum levels of IL‐6, TNF‐α, and ESR in severe patients were significantly higher than in non‐severe patients (Table 2).

**FIGURE 1:**
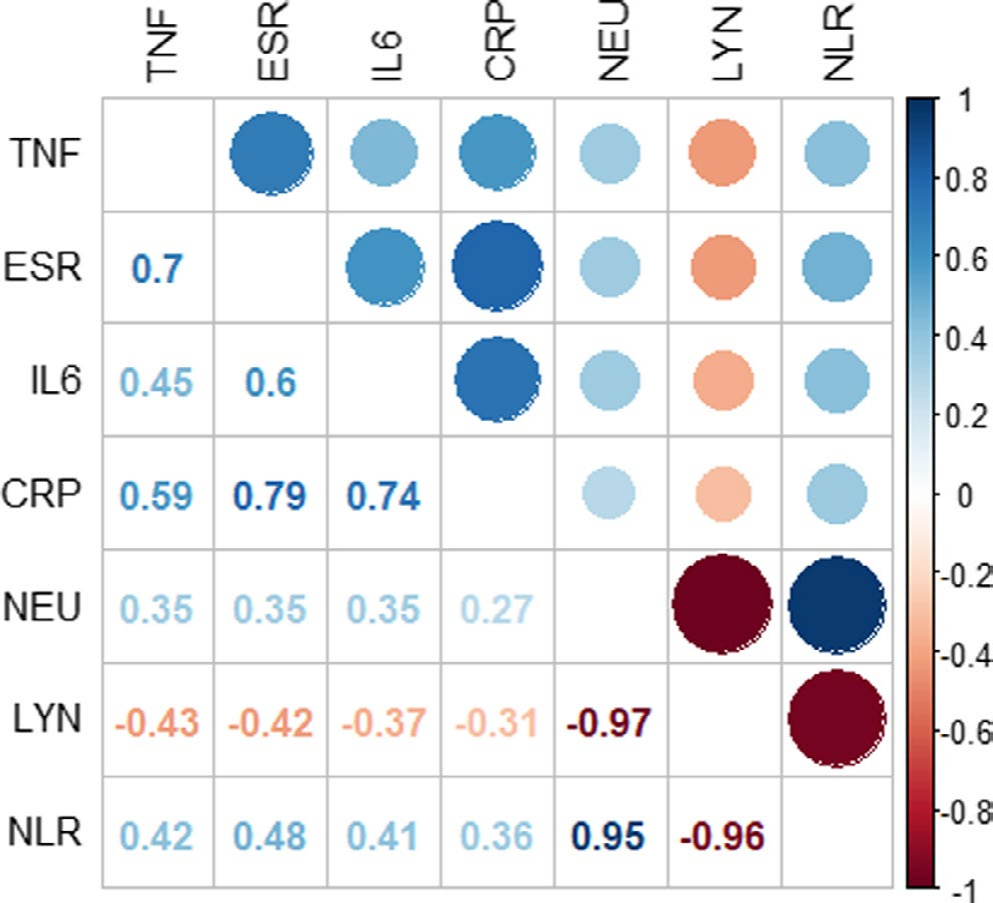
Correlation analysis between the serum laboratory parameters with severity of disease

The results of the correlation analysis between the disease severity and serum laboratory parameters are shown in Figure 1. According to the results, the correlation between IL‐6, TNFα, and CRP levels along with other factors and the disease severity was significant. There was a significant correlation between the disease severity and CRP with ESR (*r* = 0.79), CRP with IL‐6 (*r* = 0.74), LYM with NEU (*r* = −0.97), and ESR with TNF‐α (*r* = 0.7).

## 4 DISCUSSION

COVID‐19 disease is rapidly expanding worldwide. Most infected patients have mild symptoms and a good prognosis, but some of them develop severe illness that sometimes could lead to death. To date, there is no an effective therapy for COVID‐19.^15^ Therefore, it is essential to identify diagnostic markers that allow to precisely monitor the disease progression because effective early interventions are essential measures for reducing mortality. There is a great deal of evidence that suggests inflammatory responses play a critical role in the progression of COVID‐19,^16^ and several markers have the potential to be used to accurately trace and predict COVID‐19 disease severity and fatality.^17^, ^18^ Several studies have shown increased levels of cytokines in the serum of COVID‐19 patients.^19^, ^20^ Also, the effectiveness of anti‐inflammatory agents in COVID‐19 therapy highlights the critical role of inflammation in the progression of COVID‐19.^21^, ^22^, ^23^

In the present study, inflammatory markers, especially CRP, TNF‐α, and IL‐6, were found to be positively correlated with the severity of COVID‐19 by comparing these markers between the severe and non‐severe groups. Different inflammatory markers such as CRP and IL‐6 have been reported to be significantly associated with an increased risk of developing severe COVID‐19.^24^ However, these results remain controversial because some other studies have reported no significant difference in the serum levels of IL‐6, ESR, and CRP between the two groups,^25^ and the role of inflammatory parameters in monitoring COVID‐19 progression is still unclear. Similar to a study by Huang et al.,^26^ in this study, IL‐6 and TNFα levels were found to be associated with the severity of COVID‐19. Higher serum levels of pro‐inflammatory cytokines (TNF‐α and IL‐6) were found in patients with severe COVID‐19 compared to those with non‐severe disease, and these results are consistent with the results of other studies on SARS and MERS.^27^ Cytokines and chemokines are believed to play an important role in immunity and immunopathology during viral infections.^27^ Although there is no direct evidence for the involvement of inflammatory cytokines and chemokines in the lung pathology during COVID‐19, changes in laboratory parameters including high serum levels of cytokines and chemokines in infected patients have been reported to be associated with the disease severity and adverse outcomes, highlighting the potential function of hyper‐inflammatory reactions in COVID‐19 progression.^4^, ^28^

CRP is a systemic acute‐phase response marker for inflammation, infection, and tissue damage, which could be used as an inflammation indicator.^29^ Previous studies have indicated that CRP levels could be used to diagnose COVID‐19 patients and predict COVID‐19 infection outcomes.^4^, ^30^

Unlike Gao et al.,^31^ in the present study, there was no statistically significant difference in CRP levels between the non‐severe and severe groups; however, CRP levels were higher in the severe group than in the non‐severe group. Also, other studies, such as Ling^3^ and Chen et al. (2020),^32^ have reported that CRP level is positively linked to COVID‐19 severity. ESR is another inflammatory marker, which mainly reflects changes in a variety of plasma proteins.^33^ In this study, higher ESR levels were found in the severe group than in the non‐severe group, which could be attributed to the presence of more inflammation in patients with severe disease.

To the best of our knowledge, this is the first study on the association of inflammatory markers and the severity of COVID‐19 in Iran. Inflammatory markers, especially CRP, IL‐6, and TNF‐α, were significantly correlated with the severity of COVID‐19. Measurement of inflammatory markers might help clinicians monitor and evaluate the severity and prognosis of COVID‐19. Other than studies of the of cytokine as diagnostic potential, option of possible therapeutic strategy targeting either IL‐6 or IL‐10 or both is likely to emerge through analysis of such data.

## CONFLICT OF INTEREST

The authors declare that they have no conflict of interest.

## Data Availability

The data that support the findings of this study are available on request from the corresponding author.
